# Motor Imagery of Walking in People Living with and without Multiple Sclerosis: A Cross-Sectional Comparison of Mental Chronometry

**DOI:** 10.3390/brainsci11091131

**Published:** 2021-08-26

**Authors:** Douglas A. Wajda, Tobia Zanotto, Jacob J. Sosnoff

**Affiliations:** 1 Department of Health and Human Performance, Cleveland State University, Cleveland, OH 44115, USA; d.a.wajda@csuohio.edu; 2 Department of Physical Therapy, Rehabilitation Science, and Athletic Training, University of Kansas Medical Center, Kansas City, KS 66160, USA; tzanotto@kumc.edu

**Keywords:** multiple sclerosis, walking, motor imagery, dual-task, mental chronometry

## Abstract

Motor imagery represents the ability to simulate anticipated movements mentally prior to their actual execution and has been proposed as a tool to assess both individuals’ perception of task difficulty as well as their perception of their own abilities. People with multiple sclerosis (pwMS) often present with motor and cognitive dysfunction, which may negatively affect motor imagery. In this cross-sectional study, we explored differences in motor imagery of walking performance between pwMS (*n* = 20, age = 57.1 (SD = 8.6) years, 55% female) and age- and sex-matched healthy controls (*n* = 20, age = 58.1 (SD = 7.0) years, 60% female). Participants underwent mental chronometry assessments, a subset of motor imagery, which evaluated the difference between imagined and actual walking times across four walking tasks of increasing difficulty (i.e., large/narrow-width walkway with/without obstacles). Raw and absolute mental chronometry (A-MC) measures were recorded in single- (ST) and dual-task (DT) conditions. In ST conditions, pwMS had higher A-MC scores across all walking conditions (*p* ≤ 0.031, η^2^ ≥ 0.119), indicating lower motor imagery ability compared to healthy controls. During DT, all participants tended to underestimate their walking ability (3.38 ± 6.72 to 5.63 ± 9.17 s). However, after physical practice, pwMS were less able to adjust their imagined walking performance compared to healthy controls. In pwMS, A-MC scores were correlated with measures of balance confidence (ρ = −0.629, *p* < 0.01) and the self-reported expanded disability status scale (ρ = 0.747, *p* < 0.01). While the current study revealed that pwMS have lower motor imagery of walking performance compared to healthy individuals, further work is necessary to examine how the disassociation between mental chronometry and actual performance relates to quality of life and well-being.

## 1. Introduction

Humans have the innate ability to simulate anticipated movements mentally prior to the actual execution of movements [1]. This simulation is commonly termed as motor imagery (MI) and is thought to share many of the same neurocognitive structures as executed movement [2]. Indeed, multiple imaging studies have identified that imagined and executed movements activate the same regions of the motor cortex up until the movement is started [3]. Based on the ability to simulate a given movement, motor imagery has been proposed as a tool to assess both individuals’ perception of task difficulty as well as their perception of their own abilities [4].

A common method to assess mental performance is through the use of mental chronometry. Mental chronometry is a subset of motor imagery which evaluates the difference between the imagined and actual times required for the execution of a given movement, wherein smaller differences are indicative of higher motor imagery ability [5,6]. Generally, in healthy individuals, these times are similar indicating a correspondence between one’s internal models of the environment and actual capabilities [7]. For individuals with cognitive and motor impairment, however, differences in simulated performance can be an indication of a failure to update internal models or perceive and account for impairments [8] and ultimately place an individual at greater risk of falls. 

Multiple sclerosis (MS) is a disease of the central nervous system, characterized by demyelination and neurodegeneration, which affects approximately 1,000,000 people in the United States [9]. Common symptoms and limitations of people living with multiple sclerosis (pwMS) include motor and cognitive impairment [10]. Motor imagery studies seem to suggest that cognitive fatigue may adversely affect mental chronometry ability in pwMS [11,12], and, as some researchers have proposed, rehabilitation strategies involving MI may benefit both motor and cognitive function in this clinical population [13,14,15]. Nevertheless, only a few studies have examined the motor imagery of walking in pwMS [12], and it is still not clear to what extent MS impacts mental chronometry during walking tasks of increasing motor and cognitive difficulty (e.g., environmental hazards and dual-task). In addition, no studies have yet explored the relationship between motor imagery ability and measures of fall-risk and balance confidence in pwMS, both of which represent an important rehabilitation target for potential therapeutic interventions in MS [16].

The purpose of the current study was twofold. First, we aimed to explore differences in mental chronometry between people with and without MS during walking conditions of increasing difficulty. Secondly, we examined whether participants could effectively update their estimated walking performance after performing the actual walking. As a further objective, we explored the relationship between mental chronometry and measures of fall-risk and perceived balance confidence. We hypothesized that (1) pwMS would have lower motor imagery ability, as assessed through the absolute difference between imagined and actual walking times, compared to age- and sex-matched healthy individuals, and (2) pwMS would exhibit a worse performance in updating their estimated walking times after executing the actual walking tasks. In addition, we hypothesized that lower motor imagery ability would be correlated with measures of physiological fall-risk and perceived balance confidence in pwMS.

## 2. Materials and Methods

### 2.1. Study Design and Setting

A cross-sectional design was used for this study. Participants attended a laboratory-based one-hour assessment, during which they underwent motor imagery and walking while thinking tasks. Measures of demographics, mobility and cognition were also collected. For testing standardization purposes, one researcher administered all the assessments throughout the study. The study was conducted according to the ethical principles for human research, as set out by the Declaration of Helsinki. All procedures were approved by the University of Illinois Institutional Review Board (IRB #16261), and participants provided written informed consent.

### 2.2. Study Participants

A total of 40 participants were enrolled in this study. This included 20 individuals with MS and 20 age- and sex-matched healthy controls (HC). Participant recruitment was performed through a combination of online advertisements, informational flyers and local newsletters. Additionally, we recruited individuals from the local laboratory participant database. Inclusion criteria for pwMS were: (1) diagnosis of MS confirmed by a physician, (2) able to walk without bilateral support, (3) age ≥ 18 years old, (4) fluent in written and spoken English. Inclusion criteria for the control group were: (1) age ≥ 18 years old, (4) fluent in written and spoken English, (3) free from neurological diseases (e.g., epilepsy, Parkinson’s disease) and (4) no walking problems (e.g., orthopedic issues). Exclusion criteria for both groups encompassed severe cognitive impairment (score ≤ 20 on the modified telephone interview for cognitive status TICS-M [17]), pregnancy and uncorrected visual impairment.

### 2.3. Procedures

As part of the experimental set-up, participants performed four walking tasks under the following conditions: (1) walking on a large-width walkway (60 cm) without obstacles (LW), (2) walking on the large-width walkway with obstacles (5 cm wide × 2 cm high obstacles) (LW-O), (3) walking on a narrow-width walkway without obstacles (NW), (4) walking on the narrow-width walkway with obstacles (NW-O). The walkway was 7.62 m in length, and the width for the narrow-width conditions was set as 50% of the distance between the participant’s anterior superior iliac spines [18]. Participants were instructed to wear comfortable footwear during the assessments and to walk at their preferred walking speed (PWS) to execute the tasks safely. They performed two trials in single-task (ST) conditions for each walking task, in a randomized order, and the average of the two trials was taken for analysis. In addition, participants performed two additional trials for each walking task while dual-tasking (DT). The DT consisted of the serial-7 subtraction test from a given two-digit number. Participants were instructed to count backwards from the given number and complete the walking test. Participants were not instructed to focus on a specific task (i.e., walking or thinking). This DT was chosen as serial subtraction tasks have been widely utilized to quantify the cognitive-motor interaction both in healthy subjects [19] and pwMS [20]. Additionally, serial 7 s offer a consistent difficulty across multiple trials.

Prior to the walking tasks, participants underwent the mental chronometry assessment. As part of this assessment, participants sat at the starting point of the walkway and were visually presented with each walkway layout. Participants were then instructed to imagine that they are traversing the walkway from start to finish from the first-person perspective. Participants were further instructed to try to have as vivid an image in their mind as they could as if they were actually performing the task. To mirror the actual walking trials, participants were instructed to imagine walking at whatever pace they felt was appropriate to accomplish the presented walking task (i.e., PWS). Imagined trials began with a “3-2-1-Go” countdown and ended when the participants indicated reaching the end of the walkway by saying “stop”. During DT imagined movement trials, participants were given the additional instructions to imagine subtracting 7 s from a given number in their head. For consistency, all mental chronometry instructions and timing were completed by the same researcher across all subjects. The imagined time to complete all walks was recorded, and raw mental chronometry (R-MC) data were calculated as: (imagined walking time—actual walking time) across walking conditions, with positive and negative values indicating underestimation and overestimation of actual performance, respectively. The absolute values of mental chronometry (A-MC) were subsequently computed in SPSS and taken for the main analysis as the primary study outcome [21]. All motor imagery assessments were completed before the actual walking tasks and performed in the same randomized order as the actual walks. 

As a means to investigate the adjustment of perceptions, participants repeated the mental chronometry assessments following the actual walking tests. In order to avoid any testing biases, participants were not informed they would be doing a second set of imagined trials until after the actual walking tests were performed.

In addition to the imagined and actual walking tasks, participants also completed an assessment of physiological fall-risk, which was quantified through the physiological profile assessment (PPA) [22,23], and the activities-specific balance confidence (ABC) scale, as a measure of perceived balance confidence [24]. Both measures have been validated and widely utilized in MS populations [25,26,27].

### 2.4. Statistical Analysis

Statistical analyses were performed in SPSS version 26.0 (IBM, Inc., Armonk, NY, USA). The Shapiro–Wilk test was used to check whether data were normally distributed. Descriptive statistics (mean ± standard deviation or median and interquartile range) were calculated for the demographic/clinical characteristics and walking measures of interest. Independent t-tests or Mann–Whitney U tests (as appropriate) and cross-tabulation analyses were used to compare differences between pwMS and HC, for continuous and categorical variables, respectively. Differences in A-MC between pwMS and HC were analyzed by means of Mann–Whitney U tests, while differences in A-MC before and after the execution of the actual walking tasks were analyzed through Wilcoxon Signed Rank tests. A Spearman’s Rho correlation analysis was performed to investigate the relationship between A-MC and measures of physiological fall risk (PPA) and perceived balance confidence (ABC). The level of statistical significance was set at *p* ≤ 0.05.

## 3. Results

### 3.1. Study Participants 

Demographic and clinical characteristics of the study participants are summarized in Table 1. Participants were well-matched for both age (pwMS = 57.1 ± 8.6 years, HC = 58.1 ± 7 years) and sex (pwMS = 55% female, HC = 60% female). Compared to HC, pwMS had higher PPA z-scores (2 ± 1.2 vs. 0.2 ± 0.8, *p* < 0.001) and lower ABC scores (73.5 ± 20.2 vs. 92.1 ± 7.2, *p* = 0.002), which are indicative of greater physiological fall-risk and lower balance confidence, respectively.

### 3.2. Mental Chronometry

**Figure 1:**
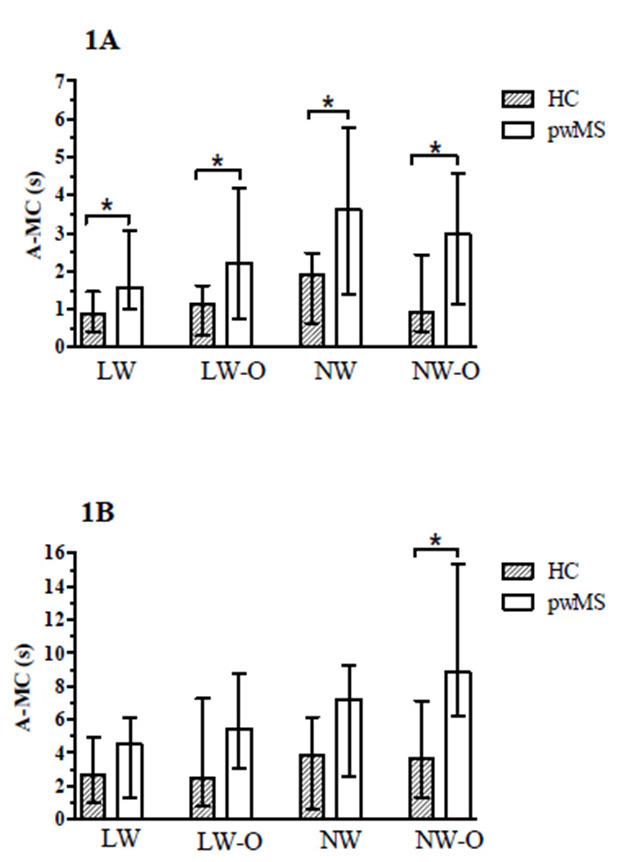
A-MC differences between pwMS and HC in ST (**1A**) and DT (**1B**) conditions. Legend: A-MC: absolute mental chronometry; HC: healthy controls; pwMS: people with multiple sclerosis; LW: large-width walkway; LW-O: large-width walkway with obstacles; NW: narrow-width walkway; NW-O: narrow-width walkway with obstacles; ST: single-task; DT: dual-task; * indicates a statistically significant difference (*p*-value ˂ 0.05).

**Figure 2:**
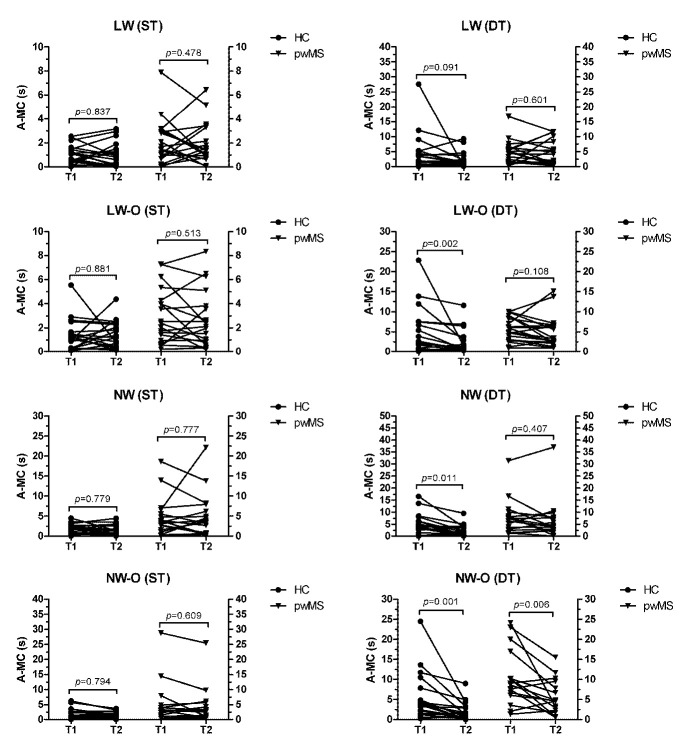
Changes in A-MC performance after actual execution of the walking tasks. Legend: A-MC: absolute mental chronometry; HC: healthy controls; pwMS: people with multiple sclerosis; LW: large-width walkway; LW-O: large-width walkway with obstacles; NW: narrow-width walkway; NW-O: narrow-width walkway with obstacles; ST: single-task; DT: dual-task; T1: trial 1 (pre-walking); T2 trial 2 (post-walking).

The mean ± standard deviation of R-MC data for each group and condition are summarized in Table 2. The Mann–Whitney U test revealed that pwMS had higher median A-MC scores compared to HC across all walking tasks (LW: 1.57(2.07)s vs. 0.87(1.03)s, *p* = 0.021, η^2^ = 0.136; LW-O: 2.22(3.43)s vs. 1.14(1.33)s, *p* = 0.031, η^2^ = 0.119; NW: 3.62(4.36)s vs. 1.91(1.84)s, *p* = 0.010, η^2^ = 0.179; NW-O: 2.98(3.42)s vs. 0.95(2.01)s, *p* = 0.017, η^2^ = 0.163) in ST conditions (Figure 1). During DT, pwMS also had higher A-MC scores for NW-O compared to HC (8.85(9.14)s vs. 3.70(5.78)s, *p* = 0.016, η^2^ = 0.167). After the execution of the actual walking trials, both groups did not improve their A-MC performance in ST conditions (Figure 2). On the other hand, HC were able improve their A-MC scores in LW-O (*p* = 0.002, z = −3.173), NW (*p* = 0.011, z = −2.539) and NW-O (*p* = 0.001, z = −3.397) following the DT walking trials, while pwMS were only able to improve A-MC performance in NW-O (*p* = 0.006, z = −2.741). The percent changes in median A-MC scores for both groups after all walking trials are summarized in Table 3.

### 3.3. Correlation Analyses

The Spearman’s Rho analysis did not reveal any significant correlations between A-MC and physiological fall-risk (PPA) in either group (Table 4). On the other hand, A-MC values during LW-O (ρ = −0.629, *p* = 0.003) and NW (ρ = −0.501, *p* = 0.034) were negatively correlated with scores on the ABC in pwMS but not in HC. In pwMS, A-MC values during LW-O (ρ = 0.747, *p* < 0.001) and NW (ρ = 0.521, *p* = 0.027) were also positively correlated with the SR-EDSS.

## 4. Discussion

In this cross-sectional study, we aimed to explore differences in mental chronometry between people with and without MS during walking tasks of increasing difficulty. In addition, we explored whether participants could effectively update their estimated walking performance, as assessed through A-MC, after performing the actual walking tasks. As we hypothesized, pwMS had higher A-MC scores compared to HC, with significant differences in all walking tasks during ST and in NW-O during DT (Figure 1). Following the actual execution of all walking tasks, no changes in A-MC during ST were detected in either group. On the other hand, in DT conditions, HC were able to decrease their A-MC scores compared to pwMS (Table 3 and Figure 2). A further objective of the current study was to examine the relationship between motor imagery and measures of fall-risk and perceived balance confidence. In this regard, the Spearman’s Rho analysis did not reveal a significant relationship between A-MC scores and the PPA, while significant negative correlations between A-MC and ABC were found in pwMS (Table 4).

A closer examination of the R-MC data suggests that, in ST conditions, both groups were overall accurate in estimating their walking performance across the different walking tasks, as indicated by scores bridging the zero value (−2.38 ± 8.61 s to 0.07 ± 2.20 s, Table 1). On the other hand, both HC and pwMS seemed to underestimate their ability during DT performance in the first trial (Table 2), which could be explained by the additional cognitive load [28]. However, examining the changes in A-MC from the pre-walking to the post-walking trial during DT, it was observed that HC were more able to update their performance, indicating a change to imagined movement based on actually performing the task. Specifically, HC exhibited significant changes in LW-O, NW and NW-O, which brought the average mental chronometry times closer to the average actual walking times. In other words, after limited physical practice of a testing condition, HC were able to update their perception of the task for improved imagined walking accuracy. Conversely, pwMS were only able to improve their A-MC performance in the most challenging condition (NW-O during DT) after they performed the walking trials. This finding seems to suggest a limited ability of pwMS to adjust internal models of a task after physical practice. Possible underlying mechanisms may involve common MS-related alterations of neural networks. Multiple sensorimotor areas, such as the supplementary motor area and the premotor, prefrontal, parietal and primary motor cortex, as well as the corticospinal tract, are thought to be activated during motor imagery of walking [29]. Since these areas are often impacted by MS [13,30], it is logical to speculate that neural connectivity disruptions may be at least partially responsible for the lower A-MC performance observed in pwMS. This seems to be indirectly corroborated by the high prevalence of motor imagery impairments in other neurological populations (e.g., Parkinson’s disease and stroke) with similar disruptions of neural networks [31]. Additionally, impairments of working memory, which are highly prevalent in MS [32,33], have also been proposed as a putative mechanism responsible for the temporal error between imagined and actual walking (i.e., mental chronometry) [34]. Importantly, some researchers have postulated that failure to update internal models may lead to over-optimistic predictions of planned actions in populations at risk of cognitive dysfunction [35].

The A-MC findings also provide valuable insight into the hazard estimate construct of the self-awareness prioritization model used to characterize the cognitive-motor interaction [36]. For instance, the observed changes in A-MC data during DT (Figure 2) suggest that HC were more likely to have a better understanding of their limits on a task after limited practice. On the other hand, pwMS may be more likely to adopt a more conservative approach to movement planning and may be willingly or unwillingly less prone to update their internal model of an action following its completion. This could possibly be viewed as a compensatory strategy to maintain safety when confronted with difficult environmental challenges. In addition, the Spearman’s Rho analysis revealed significant negative correlations between A-MC scores and measures of the hazard estimate (i.e., ABC) in pwMS but not in HC (Table 4). This could indicate a relationship between self-awareness of physical limitations and the ability to estimate walking performance, with lower balance confidence being a potential predictor of lower motor imagery ability (i.e., higher A-MC) in pwMS. Thus, the observed differences between HC and pwMS may not be exclusively attributable to neural/cognitive deficits, as commonly portrayed [21]. Indeed, our findings seem to suggest that perception of one’s own abilities may reflect the mismatch between imagined and actual walking times and be a significant driver of motor imagery ability. Further research would be required to examine in more detail whether mental chronometry assessments may be utilized to investigate self-awareness of physical disability and motor behavior in pwMS.

Despite the significant relationship with balance confidence, A-MC scores were not correlated with physiological fall-risk (PPA) in either group (Table 4). This contrasts with findings from Nakano et al. [21], who reported significant differences in mental chronometry between older adults classified at-risk or not of falls (based on one-leg stance performance). The authors concluded that an association between impaired motor imagery of walking and fall-risk may exist in elderly people [21]. On the other hand, Nilsagård et al. [37] did not find any relationship between the ability to estimate six-minute walking performance and future risk of falls in pwMS. Due to the methodological differences and different populations examined, it is not possible to compare our findings directly with those of these investigations. Thus, further research would be recommended to explore the clinical utility of mental chronometry for the assessment of fall-risk in both people living with or without MS. In this regard, it should be noted that motor imagery assessments may be easily implementable in clinical settings due to their expediency and inexpensiveness. In addition to traditional assessments of walking function and fall-risk, such as 25-foot PWS, performing a mental chronometry test could also be helpful for the identification of mental simulation impairments and to evaluate the appropriateness of therapeutic interventions. Importantly, some researchers have postulated that motor imagery exercises (visual and kinesthetic) may represent a complementary rehabilitation strategy to improve motor and cognitive outcomes through the strengthening of neural networks in pwMS [38]. Preliminary reports have highlighted the potential benefits of motor imagery interventions on measures of walking speed, fatigue and quality of life in pwMS [39]. On the other hand, it is not clear whether pharmacological interventions may positively influence motor imagery ability. Thus, further research would be recommended to explore to what extent common disease-modifying therapies may be able to prevent and/or slow down further declines of motor imagery in pwMS.

Some limitations should be acknowledged when interpreting the findings from this study. First of all, the walking tasks were performed in a controlled laboratory-based environment which limits the ecological validity of findings and their applicability to real-world settings. Secondly, we did not examine cognitive function in the current investigation. As some researchers have suggested, cognitive fatigability may play a role in the ability to update internal models, thus negatively impacting motor imagery performance in pwMS [12]. Further research would be required to elucidate this potential relationship. Lastly, it should be acknowledged that the statistical power of some analyses, as well as the overall validity of the study findings, may have been affected by the relatively small sample size.

## 5. Conclusions

This cross-sectional study revealed that pwMS have lower motor imagery of walking performance compared to HC. In addition, pwMS exhibited a lower ability to adjust the internal models of walking after physical practice, as highlighted by the smaller pre-post variation of A-MC scores following the execution of walking tasks. Lower A-MC performance was related to decreased balance confidence in pwMS. Further work is warranted to investigate the utility of mental chronometry in clinical settings, as well as its relevance to outcomes such as falls, fall-risk, community ambulation and other motor tasks.

## Figures and Tables

**Table 1:** Demographic and clinical characteristics of study participants: results are expressed as mean ± SD or median (IQR).

Variables	All Participants (40)	HC (20)	pwMS (20)	*p*-Value
Age (years)	57.6 ± 7.8	58.1 ± 7	57.1 ± 8.6	0.690
Gender (% F)	57.5	60	55	0.749
SREDSS (score)	-	-	3.8 (3.3)	N.A.
MS duration (years)	-	-	17 ± 8.3	N.A.
PPA (score)	1.1 ± 1.3	0.2 ± 0.8	2 ± 1.2	<0.001
ABC scale (score)	82.8 ± 17.7	92.1 ± 7.2	73.5 ± 20.2	0.002

Abbreviations: SD: standard deviation; IQR: interquartile range; SREDSS: Self-reported expanded disability status scale; ABC: activities-specific balance confidence; PPA: Physiological profile assessment.

**Table 2:** Raw Mental Chronometry (R-MC) data.

	LW		LW-O		NW		NW-O	
	HC	pwMS	HC	pwMS	HC	pwMS	HC	pwMS
ST								
T1	−0.65 ± 1.10	−0.68 ± 2.75	0.03 ± 1.90	−0.70 ± 3.63	0.07 ± 2.20	−1.53 ± 6.56	−0.33 ± 2.38	−2.38 ± 8.61
T2	−0.50 ± 1.30	−0.48 ± 2.50	−0.64 ± 1.66	−0.90 ± 3.41	0.05 ± 2.12	−2.54 ± 7.02	−0.75 ± 1.63	−2.90 ± 7.07
DT								
T1	4.40 ± 6.25	4.27 ± 4.14	3.38 ± 6.72	3.55 ± 5.43	3.49 ± 5.41	5.63 ± 9.17	4.40 ± 6.66	5.06 ± 11.76
T2	1.83 ± 2.78	4.34 ± 4.26	0.46 ± 3.65	3.64 ± 4.99	1.37 ± 2.94	4.22 ± 9.85	1.14 ± 2.73	0.39 ± 7.27

Abbreviations: LW: large-width walkway; LW-O: large-width walkway with obstacles; NW: narrow-width walkway; NW-O: narrow-width walkway with obstacles; HC: healthy controls; pwMS: people with multiple sclerosis; ST: single-task; DT: dual-task; T1: trial 1 (pre-walking); T2: trial 2 (post-walking). Notes: R-MC data are summarized as mean ± standard deviation of (Imagined Walk Times–Actual Walk Times).

**Table 3:** Percent change in median A-MC following execution of walking trials.

		Large-Width Walkway		Narrow-Width Walkway	
Condition	Group	LW	LW-O	NW	NW-O
ST	HC	−0.155 (↑17.9%)	+0.055 (↓4.8%)	−0.455 (↑23.8%)	+0.305 (↓32.3%)
ST	pwMS	−0.240 (↑15.3%)	−0.190 (↑8.6%)	+0.035 (↓1.0%)	−0.045 (↑1.5%)
DT	HC	−1.220 (↑46.0%)	−1.365 (↑54.9%)	−2.080 (↑53.5%)	−2.400 (↑64.9%)
DT	pwMS	−1.500 (↑33.1%)	−2.365 (↑43.4%)	−3.270 (↑45.5%)	−4.195 (↑47.4%)

Abbreviations: LW: large-width walkway; LW-O: large-width walkway with obstacles; NW: narrow-width walkway; NW-O: narrow-width walkway with obstacles; ST: single-task; DT: dual-task; HC: healthy controls; pwMS: people with multiple sclerosis; ↑ indicates improved A-MC performance; ↓ indicates decreased A-MC performance.

**Table 4:** Spearman’s Rho Correlations between A-MC and measures of physiological fall-risk and balance confidence.

		Large-Width Walkway		Narrow-Width Walkway	
	Variables	LW	LW-O	NW	NW-O
HC	PPA	−0.082	0.144	0.323	0.001
	ABC	0.124	0.037	−0.249	−0.049
	SR-EDSS	n.a.	n.a.	n.a.	n.a.
pwMS	PPA	0.080	0.408	0.342	−0.006
	ABC	−0.199	−0.629 **	−0.501 *	0.024
	SR-EDSS	0.260	0.747 **	0.521 *	0.180

Abbreviations: A-MC: absolute values of mental chronometry; LW: large-width walkway; LW-O: large-width walkway with obstacles; NW: narrow-width walkway; NW-O: narrow-width walkway with obstacles; HC: healthy controls; pwMS: people with multiple sclerosis; PPA: physiological profile assessment; ABC: activities-specific balance confidence scale; SR-EDSS: self-reported expanded disability status scale; n.a.: not applicable; * indicates a statistically significant correlation (*p*-value ˂ 0.5); ** indicates a statistically significant correlation (*p*-value ˂ 0.1).

## Data Availability

This paper utilized original data not used in other publications. The datasets generated and/or analyzed in the present study are available from the corresponding author upon reasonable request.

## References

[1] Savaki H.E., Raos V. (2019). Action perception and motor imagery: Mental practice of action. Prog. Neurobiol..

[2] Krüger B., Hettwer M., Zabicki A., de Haas B., Munzert J., Zentgraf K. (2020). Practice modality of motor sequences impacts the neural signature of motor imagery. Sci. Rep..

[3] Hardwick R.M., Caspers S., Eickhoff S.B., Swinnen S.P. (2018). Neural correlates of action: Comparing meta-analyses of imagery, observation, and execution. Neurosci. Biobehav. Rev..

[4] Ryckewaert G., Luyat M., Rambour M., Tard C., Noël M., Defebvre L., Delval A. (2015). Self-perceived and actual ability in the functional reach test in patients with Parkinson’s disease. Neurosci. Lett..

[5] Greiner J., Schoenfeld M.A., Liepert J. (2014). Assessment of mental chronometry (MC) in healthy subjects. Arch. Gerontol. Geriatr..

[6] Williams S.E., Guillot A., Di Rienzo F., Cumming J. (2015). Comparing self-report and mental chronometry measures of motor imagery ability. Eur. J. Sport Sci..

[7] Saimpont A., Malouin F., Tousignant B., Jackson P.L. (2013). Motor imagery and aging. J. Mot. Behav..

[8] Lafargue G., Noël M., Luyat M. (2013). In the elderly, failure to update internal models leads to over-optimistic predictions about upcoming actions. PLoS ONE.

[9] Wallin M.T., Culpepper W.J., Campbell J.D., Nelson L.M., Langer-Gould A., Marrie R.A., Cutter G.R., Kaye W.E., Wagner L., Tremlett H. (2019). The prevalence of MS in the United States: A population-based estimate using health claims data. Neurology.

[10] Ghasemi N., Razavi S., Nikzad E. (2017). Multiple Sclerosis: Pathogenesis, Symptoms, Diagnoses and Cell-Based Therapy. Cell J..

[11] Tacchino A., Bove M., Pedullà L., Battaglia M.A., Papaxanthis C., Brichetto G. (2013). Imagined actions in multiple sclerosis patients: Evidence of decline in motor cognitive prediction. Exp. Brain Res..

[12] Podda J., Pedullà L., Monti Bragadin M., Piccardo E., Battaglia M.A., Brichetto G., Bove M., Tacchino A. (2020). Spatial constraints and cognitive fatigue affect motor imagery of walking in people with multiple sclerosis. Sci. Rep..

[13] Heremans E., D’hooge A.M., De Bondt S., Helsen W., Feys P. (2012). The relation between cognitive and motor dysfunction and motor imagery ability in patients with multiple sclerosis. Mult. Scler..

[14] Hanson M., Concialdi M. (2019). Motor imagery in multiple sclerosis: Exploring applications in therapeutic treatment. J. Neurophysiol..

[15] Seebacher B., Kuisma R., Glynn A., Berger T. (2018). Exploring cued and non-cued motor imagery interventions in people with multiple sclerosis: A randomised feasibility trial and reliability study. Arch. Physiother..

[16] Cameron M.H., Nilsagard Y. (2018). Balance, gait, and falls in multiple sclerosis. Handb. Clin. Neurol..

[17] Bentvelzen A.C., Crawford J.D., Theobald A., Maston K., Slavin M.J., Reppermund S., Kang K., Numbers K., Brodaty H., Sachdev P. (2019). Validation and Normative Data for the Modified Telephone Interview for Cognitive Status: The Sydney Memory and Ageing Study. J. Am. Geriatr. Soc..

[18] Wong-Yu I.S., Mak M.K. (2015). Multi-dimensional balance training programme improves balance and gait performance in people with Parkinson’s disease: A pragmatic randomized controlled trial with 12-month follow-up. Parkinsonism Relat. Disord..

[19] Springer S., Giladi N., Peretz C., Yogev G., Simon E.S., Hausdorff J.M. (2006). Dual-tasking effects on gait variability: The role of aging, falls, and executive function. Mov. Disord..

[20] Gunn H., Creanor S., Haas B., Marsden J., Freeman J. (2013). Risk factors for falls in multiple sclerosis: An observational study. Mult. Scler..

[21] Nakano H., Murata S., Shiraiwa K., Nonaka K. (2020). Increased Time Difference between Imagined and Physical Walking in Older Adults at a High Risk of Falling. Brain Sci..

[22] Singh D.K., Pillai S.G., Tan S.T., Tai C.C., Shahar S. (2015). Association between physiological falls risk and physical performance tests among community-dwelling older adults. Clin. Interv. Aging..

[23] Lord S.R., Delbaere K., Gandevia S.C. (2016). Use of a physiological profile to document motor impairment in ageing and in clinical groups. J. Physiol..

[24] Powell L.E., Myers A.M. (1995). The Activities-specific Balance Confidence (ABC) Scale. J. Gerontol. A Biol. Sci. Med. Sci..

[25] Nilsagård Y., Carling A., Forsberg A. (2012). Activities-specific balance confidence in people with multiple sclerosis. Mult. Scler. Int..

[26] Hoang P.D., Baysan M., Gunn H., Cameron M., Freeman J., Nitz J., Low Choy N.L., Lord S.R. (2016). Fall risk in people with MS: A Physiological Profile Assessment study. Mult. Scler. J. Exp. Transl. Clin..

[27] Gunn H., Cameron M., Hoang P., Lord S., Shaw S., Freeman J. (2018). Relationship Between Physiological and Perceived Fall Risk in People With Multiple Sclerosis: Implications for Assessment and Management. Arch. Phys. Med. Rehabil..

[28] Leone C., Patti F., Feys P. (2015). Measuring the cost of cognitive-motor dual tasking during walking in multiple sclerosis. Mult. Scler. J..

[29] Sacheli L.M., Zapparoli L., De Santis C., Preti M., Pelosi C., Ursino N., Zerbi A., Banfi G., Paulesu E. (2017). Mental steps: Differential activation of internal pacemakers in motor imagery and in mental imitation of gait. Hum. Brain Mapp..

[30] Strik M., Chard D.T., Dekker I., Meijer K.A., Eijlers A.J., Pardini M., Uitdehaag B.M., Kolbe S.C., Geurts J.J., Schoonheim M.M. (2020). Increased functional sensorimotor network efficiency relates to disability in multiple sclerosis. Mult. Scler..

[31] Allali G., Blumen H.M., Devanne H., Pirondini E., Delval A., Van De Ville D. (2018). Brain imaging of locomotion in neurological conditions. Neurophysiol. Clin..

[32] Kouvatsou Z., Masoura E., Kiosseoglou G., Kimiskidis V.K. (2019). Working memory profiles of patients with multiple sclerosis: Where does the impairment lie?. J. Clin. Exp. Neuropsychol..

[33] Costers L., Van Schependom J., Laton J., Baijot J., Sjøgård M., Wens V., De Tiège X., Goldman S., D’Haeseleer M., D’hooghe M.B. (2021). The role of hippocampal theta oscillations in working memory impairment in multiple sclerosis. Hum. Brain Mapp..

[34] Nakano H., Murata S., Shiraiwa K., Iwase H., Kodama T. (2018). Temporal characteristics of imagined and actual walking in frail older adults. Aging Clin. Exp. Res..

[35] Beauchet O., Launay C.P., Sejdić E., Allali G., Annweiler C. (2014). Motor imagery of gait: A new way to detect mild cognitive impairment?. J. Neuroeng. Rehabil..

[36] Yogev-Seligmann G., Hausdorff J.M., Giladi N. (2012). Do we always prioritize balance when walking? Towards an integrated model of task prioritization. Mov. Disord..

[37] Nilsagård Y., Westerdahl E., Wittrin A., Gunnarsson M. (2016). Walking Distance as a Predictor of Falls in People With Multiple Sclerosis. Physiother. Res. Int..

[38] Seebacher B., Kuisma R., Glynn A., Berger T. (2017). The effect of rhythmic-cued motor imagery on walking, fatigue and quality of life in people with multiple sclerosis: A randomised controlled trial. Mult. Scler..

[39] Gil-Bermejo-Bernardez-Zerpa A., Moral-Munoz J.A., Lucena-Anton D., Luque-Moreno C. (2021). Effectiveness of Motor Imagery on Motor Recovery in Patients with Multiple Sclerosis: Systematic Review. Int. J. Environ. Res. Public Health.

